# Molecular epidemiology and genetic diversity of infectious bronchitis virus in Iranian poultry: A systematic review

**DOI:** 10.1016/j.psj.2026.107786

**Published:** 2026-09-16

**Authors:** Arash Ghalyanchi Langeroudi, Hamideh Najafi, Kimia Malekahmadi, Sayyad Khanizadeh, Iman Pouladi

**Affiliations:** a Department of Microbiology and Immunology, Faculty of Veterinary Medicine, University of Tehran, Iran; b Department of Pathobiology, Faculty of Veterinary Medicine, Lorestan University, Khorramabad, Iran; c Hepatitis Research Center, Virology Group, School of Medicine, Lorestan University of Medical Sciences, Khorramabad, Iran

**Keywords:** Avian coronavirus, GI-23, S1 gene, Molecular epidemiology, Infectious bronchitis virus

## Abstract

Infectious bronchitis virus (**IBV**) is a highly contagious avian coronavirus that causes substantial economic losses in poultry production due to respiratory disease, reduced performance, and impaired egg production. In Iran, IBV has been repeatedly reported over the past two decades; however, evidence on diagnostic approaches and circulating lineages remains fragmented. This systematic review aimed to summarize the available Iranian literature on the molecular epidemiology and genetic diversity of IBV in poultry. We conducted a comprehensive search for relevant papers that reported the identification or molecular characterization of infectious bronchitis virus in Iranian poultry using key scientific databases and associated resources. We included 32 studies published between 2003 and 2022. Most investigations were conducted in broiler flocks, and most studies used molecular diagnostic methods, with RT-PCR being the most frequently reported approach, followed by real-time RT-PCR and nested RT-PCR; ELISA was used in a limited number of studies. Diverse circulating lineages were reported; GI-1 (Massachusetts-type), GI-13 (793/B-type), GI-19 (QX-like), and GI-23 (Variant-2/IS-1494/06-like) strains were often documented, suggesting co-circulation of many lineages in Iranian poultry populations. Genotype reporting was inconsistent across studies, and several investigations lacked complete molecular characterization. Additionally, quantitative synthesis of prevalence estimates could not be performed because the extracted dataset did not consistently report denominators (sample size) and numbers of positive samples. Overall, the evidence suggests sustained circulation of genetically diverse IBV lineages in Iran, supporting the need for continuous molecular surveillance, harmonized reporting, and vaccination strategies aligned with locally circulating strains to improve control of infectious bronchitis in Iranian poultry.

## Description of problem

Infectious bronchitis virus (**IBV**) belongs to the family Coronaviridae. IBV is a highly contagious avian Gammacoronavirus primarily affecting chickens, although it can also circulate among other bird species (Quinteros et al., 2022; Bande et al., 2017; Falchieri et al., 2024). Infectious bronchitis (**IB**) ranks among the most contagious avian viral diseases and, following highly pathogenic avian influenza, represents the second leading cause of economic losses in countries with well-established poultry industries worldwide (De Wit et al., 2011; Ramakrishnan et al., 2019). Poultry breeders face significant financial strain due to the acute and highly contagious respiratory infection known as infectious bronchitis virus (**IBV**). The virus constantly reemerges in many serotypes and genotypes in a variety of geographical locations and displays varying tissue tropism. IBV can replicate in different organs and target the respiratory, reproductive, or urinary systems, depending on the strain (Bhuiyan et al., 2021; Abozeid, 2023). IB can present as respiratory, renal, or reproductive forms, depending on the infecting strain and the affected organ system (Abozeid, 2023). The most common symptoms are respiratory, such as nasal discharge, sneezing, coughing, and tracheal rales; renal involvement presents as nephritis and urate deposits; and reproductive variants result in reduced egg production and abnormalities in egg quality. In cases worsened by secondary bacterial infections such as Escherichia coli and Mycoplasma spp, which can cause caseous lesions in the trachea, pericardium, and air sacs, mortality is typically low but can rise to 20–30% (Motamed et al., 2022; Abozeid, 2023). Avian infectious bronchitis is an extremely contagious disease that can rapidly spread within chicken flocks (Zhang et al., 2021). Continuous monitoring and characterization of circulating IBV strains in a given region are essential to implement the most effective control strategies. Interspecies transmission has been documented within this virus family, highlighting the importance of understanding IBV pathobiology to anticipate better the emergence of new serotypes. Several IBV genotypes are prevalent in chicken populations worldwide. Complete sequencing of the S1 gene, regarded as the gold standard for strain identification and phylogenetic categorization, underpins the most dependable method for IBV genotyping (Valastro et al., 2016). Based on phylogenetic analysis of full S1 gene sequences, this method has led to a widely recognized nomenclature that defines six genotypes (GI–GVI), including 32 different viral lineages (Valastro et al., 2016). The trachea, kidneys, oviduct, lungs, esophagus, proventriculus, gut, liver, spleen, bursa, cecal tonsils, cloaca, and testes are just a few of the organs that the virus itself can attack (Ghalyanchilangeroudi et al., 2023). Many IBV genotypes have been reported in Iran. Seven different genotypes—Massachusetts (Mass), 793/B, IS720, Variant 2 (IS-1494-like), QX, IR-I, and IR-II—were found to be present in Iranian chicken farms during the first thorough investigation carried out between 2010 and 2014 (Hosseini et al., 2015). Other lineages have since been identified, such as D274 (Ghalyanchilangeroudi et al., 2019), Q1 (Ghalyanchilangeroudi et al., 2020), and D1466 (Ghalyanchilangeroudi et al., 2020). The currently circulating lineages in Iran are primarily categorized under genotype GI, which includes GI-1 (Massachusetts), GI-13 (793/B), GI-19 (QX), and GI-23 (Variant 2/IS-1494/06-like), according to the globally recognized classification system (Valastro et al., 2016). Research indicates that while the prevalence of other genotypes has declined, the GI-23 (Variant 2) genotype has become more common in recent years. A thorough understanding of the predominant IBV genotypes in a region enables the use of rapid diagnostic techniques and optimal control strategies, thereby minimizing mortality and reducing the economic impact of this highly contagious viral disease. Therefore, this work aims to review infectious bronchitis virus (**IBV**) in Iranian poultry, including its genetic epidemiology, diagnostic methods, geographic distribution, circulating IBV lineages, and information gaps.

## Materials and methods

### Data extraction and search strategy

We conducted a systematic search using terms including “avian infectious bronchitis virus,” “AIBV,” “avian coronavirus,” “broiler chickens,” “broiler flocks,” and “Iran.” We thoroughly searched English-language databases such as MEDLINE, Google Scholar, PubMed, Scopus, ScienceDirect, and Web of Science (ISI), as well as Persian databases including Iran Doc, Iran Medex, Magiran, and SID. For the Google Scholar search, the first 200 records were screened by relevance ranking. A stopping rule was applied whereby the search was terminated when two consecutive pages yielded no new relevant studies. Google Scholar was used as a supplementary source to identify additional studies that the primary databases may not have captured. The systematic literature search was performed on March 15, 2023. To ensure comprehensive coverage, we examined abstracts and summaries from congresses alongside published studies. We reviewed both Persian and English articles and independently assessed them to establish a standardized data extraction protocol.

Most included studies (around 85%) did not consistently record both the denominator (total sample size) and numerator (number of positive samples) in a standardized, extractable manner, as became clear during data extraction. Additionally, some studies presented unclear denominators, while others reported only the number of positive flocks without reporting the total number of flocks sampled. In accordance with the Preferred Reporting Items for Systematic Reviews and Meta-Analyses (**PRISMA**) criteria for systematic reviews without meta-analysis, we restricted our synthesis to a narrative explanation of the findings, as we could not conduct a meta-analysis.

We treated each included study as a single unit of analysis to summarize genotype distribution. When a research report reported more than one genotype, we counted each genotype independently and tallied the study once for each genotype. Therefore, rather than the quantity of individual viral detections or isolates, the proportionate distribution of genotypes represents the frequency of reporting at the study level. We selected this method because we could not calculate detection-level prevalence, as most included studies did not report the number of positive samples per genotype.

### Study selection and identification

As illustrated in Fig. 1, our search initially identified 140 articles addressing the prevalence and associated factors of avian infectious bronchitis virus (**IBV**) and related diseases in Iran. After removing 32 duplicate records, 108 studies remained. Of these, 29 were excluded based on the title alone, and 19 were further excluded following abstract review based on the predefined eligibility criteria (e.g., non-original data, case reports, serology-only studies, or studies focusing on non-chicken species). We conducted full-text assessments of the remaining 60 articles to evaluate eligibility according to the inclusion and exclusion criteria described above. Ultimately, 32 studies met all criteria and were included in the final systematic review (Fig. 1).

**Fig. 1:**
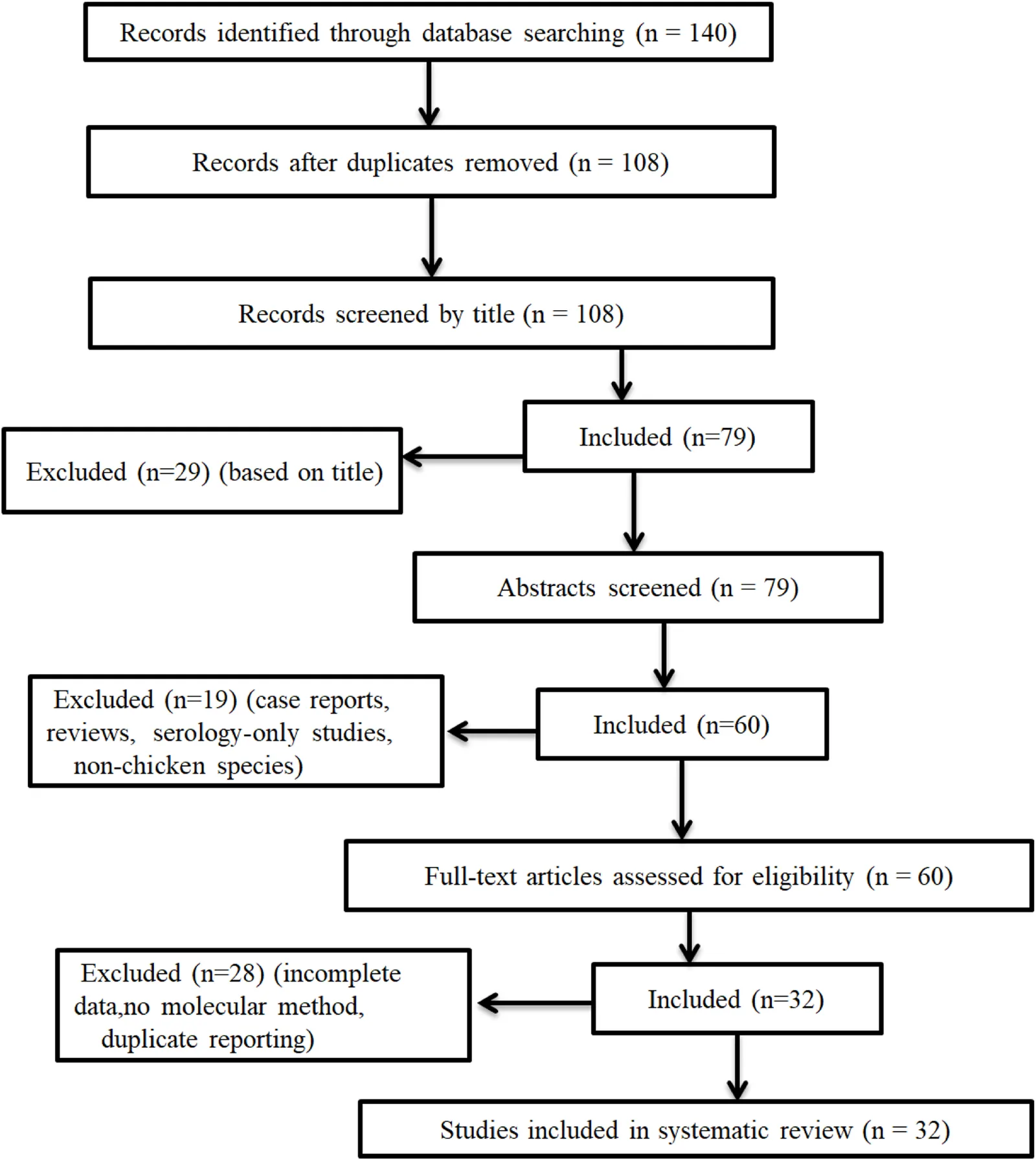
Flow diagram illustrating the selection process of studies included in the systematic review on the prevalence and associated factors of avian infectious bronchitis virus in Iran.

### Study inclusion and exclusion criteria

We included studies focused on chickens in Iran, including broilers, layers, backyard, and mixed-flock populations, published in English or Persian between January 2003 and December 2022. They must report genetic characterization methods such as S1 gene sequencing, phylogenetic analysis, and genotyping or molecular detection methods such as RT-PCR, real-time RT-PCR, and nested RT-PCR for IBV. We excluded case reports, narrative or systematic reviews, meta-analyses, editorials, conference abstracts without full text, letters to the editor, opinion pieces, studies that used only serological detection (e.g., ELISA) without molecular confirmation, and studies with insufficient data to allow meaningful extraction. Additionally, studies that solely concentrated on non-chicken avian species or did not provide original data were not included.

### Quality assessment

Two reviewers independently evaluated the methodological quality of the included studies using a standardized checklist that was modified from the Newcastle-Ottawa Scale (**NOS**) for cross-sectional studies and the Joanna Briggs Institute (**JBI**) Critical Appraisal Checklist for Prevalence Studies. Clarity of study objectives, suitability of sampling strategy, adequacy of sample size in relation to the study population, validity and reliability of the diagnostic method (e.g., RT-PCR, real-time RT-PCR, nested RT-PCR, or ELISA), completeness of molecular characterization including S1 gene sequencing and phylogenetic analysis, and transparency and clarity of reporting were among the evaluation criteria. A score of "yes" (met), "no" (not met), or "unclear" (insufficient information) was assigned to each criterion. Based on the overall score, studies were classified as high, moderate, or poor quality. Reviewers resolved disagreements through discussion or consultation with a third reviewer.

### Protocol registration

The protocol for this systematic review was not registered in PROSPERO or the Open Science Framework (**OSF**) before conducting the review. Although the review followed the PRISMA (Preferred Reporting Items for Systematic Reviews and Meta-Analyses) guidelines for systematic reviews without meta-analysis, we acknowledge the lack of prospective protocol registration as a limitation. We addressed this limitation in the Discussion section.

## Results and discussion

Overall, the data show the widespread use of PCR-based assays for IBV identification and highlight frequent reports of several IBV lineages in Iran. Most of the 32 included studies used molecular diagnostic techniques: the most commonly reported method was conventional RT-PCR, used in 18 studies (56.25%), followed by real-time RT-PCR in 8 studies (25.00%) and nested RT-PCR in 5 studies (15.62%). Only two investigations (6.25%) revealed serological detection using ELISA. The complementary roles of molecular and serological methods in IBV monitoring are shown by numerous investigations that used multiple diagnostic techniques. For lineage reporting, 26 studies (81.25%) reported molecular characterization data, whereas the remaining 6 studies (18.75%) reported IBV detection without genotyping. These findings emphasize ongoing circulation of genetically diverse strains and support the need for harmonized molecular surveillance and standardized reporting across Iranian poultry studies. Studies reported genetic diversity and genotype patterns inconsistently.

Of the 32 included papers, 26 (81.25%) carried out molecular characterization, while 6 (18.75%) reported IBV detection without genotyping. GI-1 (Massachusetts-type), which was reported in 14 studies (43.75%); GI-13 (793/B-type) in 13 studies (40.62%); GI-19 (QX-like) in 12 studies (37.50%); and GI-23 (Variant-2/IS-1494/06-like) in 10 studies (31.25%). Other IBV lineages, albeit with much lower reporting rates, have also been found in Iranian poultry in addition to these four main lineages. These include GI-16 (IS-720), which was reported in three studies (9.37%) carried out between 2015 and 2020, mostly in multi-province surveillance efforts; IR-1 and IR-2, which are native Iranian genotypes found in two studies each (6.25%) and seem to be geographically restricted to particular areas, potentially representing distinct evolutionary lineages within the Iranian poultry population; and GI-8 (D274), D1466, and Q1, each reported in a single study (3.12%). Ghalyanchilangeroudi et al. (2019) found the D274 lineage, traditionally linked to IBV outbreaks in Europe, in broiler flocks, indicating that this lineage may have been introduced or emerged in the area. The Q1 lineage, initially identified in China and linked to respiratory and renal disorders, was found in northern Iran (Ghalyanchilangeroudi et al., 2020), suggesting potential transboundary spread, while the D1466 lineage was reported in a retrospective analysis spanning 2013–2017 (Ghalyanchilangeroudi et al., 2020). Low prevalence, geographic limitations, sample biases, changes in surveillance intensity among provinces, or discrepancies in diagnostic techniques and sequencing processes across studies may have contributed to the poor reporting of these new lineages. However, their discovery highlights the significant genetic diversity of IBV in Iran and the importance of thorough S1 gene sequencing to capture the range of circulating strains fully. While the frequency of other genotypes has decreased over the same period, the majority of studies reporting GI-23 (Variant-2/IS-1494/06-like) strains were published after 2015, indicating an increasing prevalence or recognition of this lineage in recent years (Hamadan et al., 2017; Ghalyanchilangeroudi et al., 2019; Motamed and Bashashati, 2022). Because denominator values were not consistently provided across studies, these proportions indicate study-level reporting frequencies (Fig. 2) rather than the true frequency of each genotype.

**Fig. 2:**
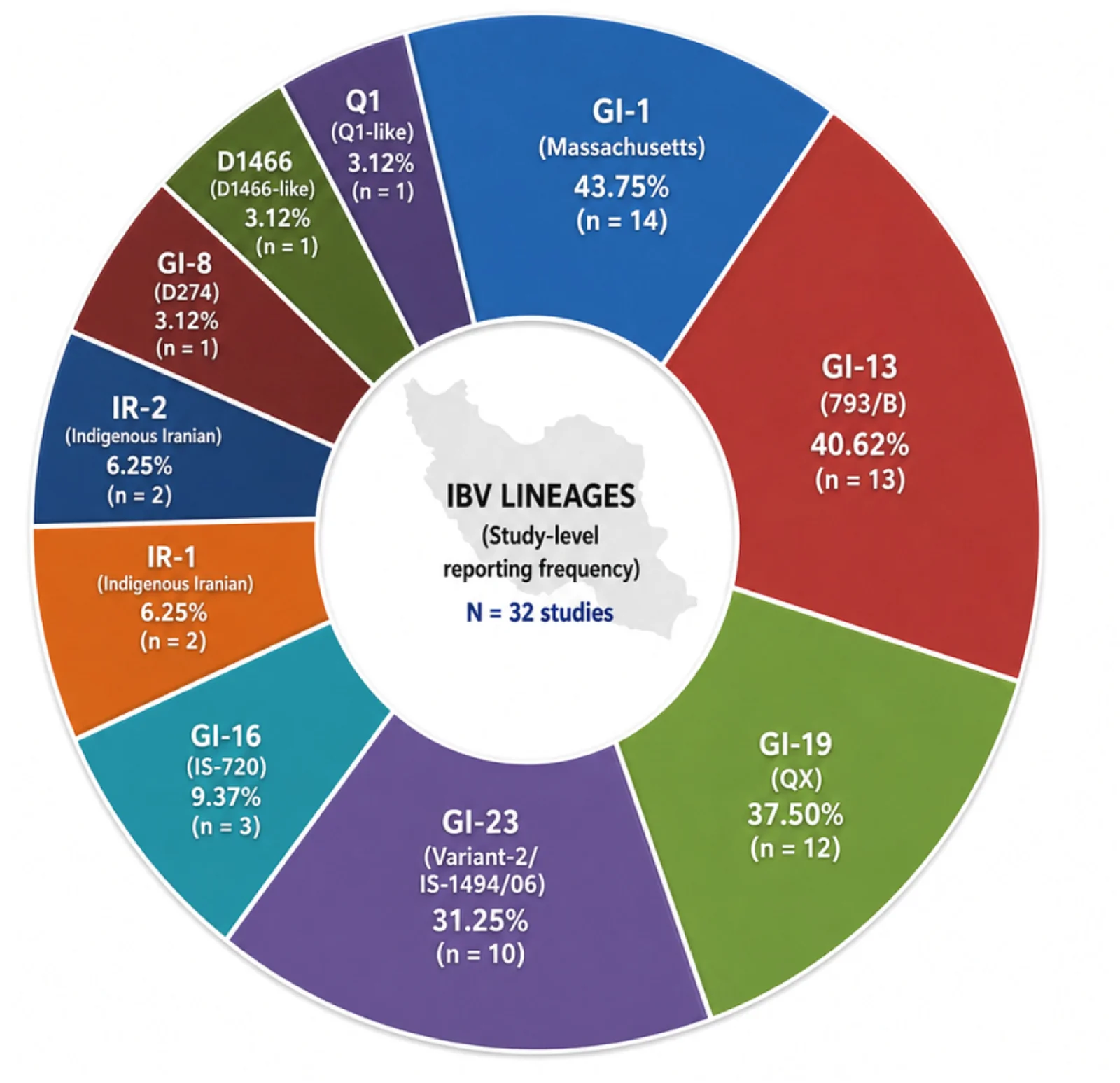
Proportional distribution of reported IBV serotype/genotype groups across included studies. The denominator is the total number of included studies (N = 32), and each study was counted once for each genotype it reported. Therefore, the percentages represent the proportion of studies reporting each genotype (study-level reporting frequency), not the number of individual virus detections or the prevalence of each genotype in the poultry population. The most frequently reported groups were Massachusetts-type (n = 14 studies, 43.75%), 793/B-type (n = 13 studies, 40.62%), QX-like (n = 12 studies, 37.50%), and Variant-2/IS-1494/06-like (n = 10 studies, 31.25%). Other genotypes (IS-720, IR-1, IR-2, D274, D1466, Q1) were reported in fewer than 5 studies each.

For several interrelated reasons, the GI-23 (Variant-2/IS-1494/06-like) lineage has become the most common Infectious Bronchitis Virus (**IBV**) strain in Iran. The minimal genetic homology found between GI-23 field isolates and the widely used vaccines in Iran, namely H120 and 4/91, is crucial. According to research, nucleotide homology between these vaccines and Iranian GI-23 isolates varies from 82.35% to 83.13%, but Massachusetts-type field isolates exhibit even lower homology, from 71.80% to 75.30% (Motamed and Bashashati, 2022). According to Hamadan et al. (2017) and Ghalyanchilangeroudi et al. (2019), this significant antigenic disparity suggests that current vaccination strategies are ineffective, failing to provide adequate cross-protection against GI-23, which facilitates viral circulation and outbreaks in vaccinated poultry (Hamadan et al., 2017; Ghalyanchilangeroudi et al., 2019). Recombination events have also significantly influenced the development of the GI-23 lineage. Complete genomic analyses of an Iranian GI-23 isolate (named Mahed) show a mosaic genomic structure made up of several lineages, with some parts grouping with QX-type strains and others with Q1 strains (Mousavi et al., 2018). This recombination improves GI-23′s genetic diversity and adaptability. Recent research has shown that new mutations have emerged within this lineage, including synonymous mutations (T954C and G1056A) and a non-synonymous mutation (C797T) that is exclusive to isolates from Iran. According to Ghalyanchilangeroudi et al. (2023), these alterations may improve the virus's fitness, transmissibility, or capacity to elude immunological responses. Furthermore, GI-23 was first discovered in the Middle East in 1998, and more recent research indicates it may have also migrated to Europe (Fan et al., 2022; Valastro et al., 2016). This worldwide spread increases the risk of co-infections and further recombination, which might lead to the emergence of novel variations. In conclusion, low vaccine homology, recombination-driven evolution, continuous mutational diversity, and wide geographic dispersion contribute to the prevalence of GI-23 in Iranian poultry (Hamadan et al., 2017; Ghalyanchilangeroudi et al., 2019; Motamed and Bashashati, 2022).

Overall, these results demonstrate the crucial role of PCR-based approaches in molecular epidemiological research and IBV detection in Iran, in line with global practice, where molecular assays offer higher analytical sensitivity and allow lineage assignment compared with serological assays alone. Broiler flocks were the most commonly studied poultry population among the included studies, whereas a smaller percentage assessed backyard/village hens, broiler–layer mixed populations, or multiple flock types (Table 1). Geographically speaking, studies were carried out in several provinces and multi-province contexts, indicating that IBV research has not been restricted to a particular area. Geographic reporting, however, was inconsistent; in several studies, the sample region was referred to as "Iran" or comprised several provinces (Table 1).

**Table 1:** Characteristics of included studies (N = 32).

Row	Year	Province/Region	Poultry type	Diagnostic method	Reported lineage(s)	Sample size (total)	Number positive	Detection rate (%)	Resources
1	2003	Fars	Broiler	RT-PCR	GI-13 (4/91), GI-1 (Massachusetts)	113	69	61.1	Shahriari et al., 2003
2	2004	16 provinces	Broiler	Nested RT-PCR	GI-13 (4/91)	NR	NR	N/A	Seyfi Abad Shapouri et al., 2004
3	2006	Esfahan	Backyard/Village	ELISA	Not applicable (serology only)	360	108	30.0	Mahzounieh et al., 2006
4	2008	13 provinces	Mixed/Multiple	Nested RT-PCR	GI-1 (Massachusetts), GI-13 (793/B)	NR	NR	N/A	Pourbakhsh et al., 2008
5	2011	Southwestern Iran	Broiler	ELISA	Not applicable (serology only)	NR	NR	N/A	Hadipour et al., 2011
6	2012	Fars	Broiler	Nested RT-PCR	GI-13 (4/91), GI-1 (Massachusetts)	210	48	22.9	Poorbaghi et al., 2012
7	2013	Zabol	Broiler	RT-PCR	GI-1 (Massachusetts)	192	68	35.4	Jahantigh et al., 2013
8	2016	8 provinces	Broiler	Nested RT-PCR	GI-23 (IS-1494-like), GI-13 (793/B), GI-1 (Massachusetts), GI-16 (IS-720), GI-19 (QX)	NR	NR	N/A	Najafi et al., 2016
9	2015	Iran	Mixed/Multiple	RT-PCR	GI-1 (Mass), GI-13 (793/B), GI-16 (IS-720), GI-23 (Variant 2), GI-19 (QX), IR-I, IR-II	NR	NR	N/A	Hosseini et al., 2015
10	2016	Azarbaijan, Sistan, Qazvin	Broiler	RT-PCR	GI-19 (QX)	NR	NR	N/A	Davam et al., 2016
11	2016	Iran	Broiler & layer	RT-PCR	GI-1 (Massachusetts), GI-13 (793/B), GI-23 (Variant 2)	NR	NR	N/A	Homayounimehr et al., 2016
12	2017	Tehran	Chicken (unspecified)	Real-time RT-PCR	IR-1	NR	NR	N/A	Najafi et al., 2017
13	2017	8 provinces	Broiler	Real-time RT-PCR	GI-23 (IS-1494-like), GI-13 (793/B), GI-19 (QX), GI-1 (Massachusetts)	404	207	51.2	Hamadan et al., 2017
14	2017	Bushehr	Broiler	RT-PCR	GI-1 (Massachusetts), GI-23 (Variant 2)	NR	NR	N/A	Saadat et al., 2017
15	2017	15 provinces	Broiler	RT-PCR	GI-13 (793/B)	NR	NR	N/A	Yousefzadeh Kalokhoran et al., 2017
16	2018	Ahvaz	Backyard/Village	RT-PCR	Not reported (detection only)	320	124	38.8	Boroomand et al., 2018a
17	2018	Iran	Broiler & layer	Real-time RT-PCR	GI-13 (793/B), GI-19 (QX-like)	NR	NR	N/A	Masoudian et al., 2018
18	2018	Iran	Broiler	Other	GI-23 (IS-1494/06)	NR	NR	N/A	Mousavi et al., 2018
19	2018	Mazandaran	Backyard/Village	RT-PCR	GI-13 (793/B), GI-23 (Variant 2), GI-19 (QX)	NR	NR	N/A	Shokri et al., 2018
20	2018	Iran	Broiler	Nested RT-PCR	Not reported (detection only)	NR	NR	N/A	Gholami et al., 2018
21	2018	Ahvaz	Broiler	RT-PCR	GI-19 (QX)	48	22	45.8	Boroomand et al., 2018b
22	2019	31 provinces	Broiler	Real-time RT-PCR	GI-23 (Variant 2), GI-13 (793/B)	NR	NR	N/A	Haji-Abdolvahab et al., 2019
23	2021	Mashhad	Broiler & layer	Nested RT-PCR	Not reported (detection only)	264	84	31.8	Tabatabaeizadeh et al., 2021
24	2019	Iran	Broiler	Real-time RT-PCR	GI-8 (D274)	420	60	14.3	Ghalyanchilangeroudi et al., 2019
25	2020	North of Iran	Broiler	RT-PCR	Q1	NR	NR	N/A	Ghalyanchilangeroudi et al., 2020a
26	2019	Tehran, Gilan, Ardabil	Broiler	RT-PCR	GI-13 (793/B), GI-1 (Massachusetts)	NR	NR	N/A	Salarpour et al., 2019
27	2020	Gilan	Mixed/Multiple	RT-PCR	GI-19 (QX), GI-23 (IS-1494)	NR	NR	N/A	Rezaee et al., 2020
28	2020	IR-1, IR-2	Broiler & layer	Real-time RT-PCR	GI-1 (Massachusetts), GI-19 (QX), GI-16 (IS-720), GI-23 (IS-1494), GI-13 (793/B), IR-1, IR-2	NR	NR	N/A	Ghalyanchilangeroudi et al., 2020b
29	2020	4 provinces	Broiler	RT-PCR	Not reported (detection only)	NR	NR	N/A	Ghorbiani et al., 2020
30	2021	Golestan	Broiler	RT-PCR	GI-19 (QX)	210	68	32.4	Montazeri et al., 2021
31	2022	4 provinces	Broiler	RT-PCR	GI-23 (IS-1494), GI-1 (Massachusetts)	NR	NR	N/A	Motamed and Bashashati, 2022
32	2021	Golestan, Mazandaran	Broiler	Real-time RT-PCR	Not reported (detection only)	NR	NR	N/A	Hajijafari Anaraki et al., 2021

This systematic review included 32 studies investigating the molecular detection and/or genetic characterization of infectious bronchitis virus (**IBV**) in Iranian poultry. The included publications spanned from 2003 to 2022, indicating sustained research activity on IBV molecular epidemiology in Iran. Table 1 summarizes the characteristics of all included studies.

Researchers in Iran identified four pathotype groups—respiratory, nephropathogenic, reproductive, and mixed in circulating IBV lineages. Respiratory symptoms were the primary IBV symptom in broiler flocks in 68.75% of the 32 trials. In 28.12% of investigations, nephropathogenic strains associated with QX-like and Variant-2/IS-1494/06-like lineages were identified; these strains frequently resulted in death from renal problems. Reduced egg yield and quality were among the reproductive issues linked to Massachusetts-type and 793/B-type strains in layer flocks. 18.75% of investigations found mixed pathotypes impacting several systems, demonstrating the ability of certain Iranian lineages to have a variety of tissue tropisms.

The current systematic analysis shows that IBV research has been ongoing and geographically extensive in Iran, with a primary focus on commercial broiler production systems, reflecting the sector's economic significance. Although the prevalence of PCR-based diagnostic techniques is consistent with global practice, direct comparisons between studies are limited due to variations in sampling procedures and targeted regions (Legnardi et al., 2020). Crucially, the continued circulation of GI-1, GI-13, and GI-19, together with the recurrent identification of GI-23 as the main lineage in recent years, illustrates the critical requirement for frequent vaccine–field strain matching and the shortcomings of monovalent immunization regimens. The sporadic detection of additional lineages (GI-16, IR-1, IR-2, GI-8, D1466, and Q1) further emphasizes the substantial genetic diversity of IBV in Iran. It suggests that some of these may be geographically restricted or under‑sampled due to surveillance biases.

Approximately 94% of the included studies had moderate to high methodological quality, as assessed using the JBI Critical Appraisal Checklist and the Newcastle-Ottawa Scale. This was largely due to validated molecular diagnostic techniques such as RT-PCR and S1 gene sequencing. Methodological issues were identified, including imprecise reporting of sampling procedures, inadequate sample size justification, and incomplete molecular characterization in certain investigations. The interpretation of circulating lineages in this systematic review may be affected because only 12 studies (37.5%) provided sufficient molecular data for accurate lineage assignment. A key finding of this review is the repeated reporting of multiple IBV lineages, including GI-1 (Massachusetts), GI-13 (793/B), GI-19 (QX-like), and GI-23 (Variant-2/IS-1494/06-like), across Iranian poultry. Global reviews indicate that these lineages have broad geographic distribution and that the emergence of new variants is driven by mutation and recombination, creating ongoing challenges for control. From a practical standpoint, the co-circulation of antigenically and genetically distinct lineages can reduce the effectiveness of single-strain vaccination programs and highlights the need for continuous molecular surveillance and periodic evaluation of vaccine field strain matching (Valastro et al., 2016; Fan et al., 2022). Vaccine control attempts are complicated by the co-circulation of many Infectious Bronchitis Virus (**IBV**) lineages in Iran. Although Massachusetts-type vaccines provide low cross-protection against heterologous lineages, such as QX-like and Variant-2/IS-1494/06-like strains, they give robust protection against related field strains (de Wit et al., 2011; Gallardo, 2021). Evidence suggests that 793/B-type vaccinations may provide some protection against certain variations, although their effectiveness varies. According to Abbas et al. (2022) and Bhuiyan et al. (2021), the Variant-2/IS-1494/06-like lineage, more often observed in Iran, is antigenically distinct from other lineages, resulting in less-than-ideal vaccination protection (Abbas et al., 2022; Bhuiyan et al., 2021). Therefore, immunization plans should consider combination vaccines or region-specific vaccines designed for dominant lineages. Future research must continuously monitor antigenic matching using molecular characterization and serological tests to fill current knowledge gaps.

Iranian poultry sector now uses several approved IBV vaccine strains. The most often used vaccinations are those of the Massachusetts type, such as H120, H52, and Ma5, which are frequently given as inactivated vaccines to breeders and layers and as live attenuated vaccines to broilers (Hamadan et al., 2017; Ghalyanchilangeroudi et al., 2019). Furthermore, combination vaccination programs increasingly use 793/B-type vaccines, such as commercial 4/91 and IB88 strains and the native Razi 793/B.08IR strain (a live attenuated vaccine developed specifically for Iranian field isolates) (Ghalyanchilangeroudi et al., 2023; Pourbakhsh et al., 2008). The Razi strain has demonstrated high nucleotide identity (98-100%) with both locally used commercial vaccines and Iranian 793/B-like field isolates, suggesting its continued relevance for protection against circulating 793/B variants (Ghalyanchilangeroudi et al., 2023; Shokri et al., 2018).

Despite extensive immunization, IBV outbreaks continue in Iran for several reasons. According to cross-neutralization investigations, antisera from vaccinations such as H120, H52, and 4/91 are ineffective against current Iranian genotypes, including IS-1494, IS-720, and IR-1, suggesting notable antigenic changes (Hamadan et al., 2017; Najafi et al., 2016). Furthermore, Variant-2 exhibits only 82.35% to 83.13% identity with the 4/91 and H120 vaccinations, indicating low amino acid homology between Iranian field isolates and vaccine strains (Motamed and Bashashati, 2022). As seen in a 2020–2021 epidemic in which 84% of sick flocks had received vaccinations but were still vulnerable to Variant-2, this mismatch leads to low vaccine efficiency (Haji–Abdolvahab et al., 2019; Motamed and Bashashati, 2022).

In coronaviruses like IBV, recombination plays a major role in genetic diversity and evolution, which is connected to the worldwide development of novel genotypes and serotypes (Valastro et al., 2016; Fan et al., 2022). A knowledge gap remains because, despite the presence of several IBV lineages in Iran, no research has documented recombination events. The co-circulation of lineages such as Massachusetts, 793/B, QX-like, and IS-1494/06-like in Iran suggests the possibility of recombination, especially with the rise of the QX-like and IS-1494/06-like lineages. To identify recombination breakpoints and understand IBV's evolutionary dynamics, future research should concentrate on whole-genome sequencing of Iranian IBV strains and complete S1 gene sequencing. This will help differentiate recombination from mutation-driven evolution (Mousavi et al., 2018; Legnardi et al., 2020).

Evidence from nearby nations supports the importance of backyard poultry in IBV maintenance. Research from Iraq reported a 27.5% frequency of respiratory symptoms in backyard hens (Al-Ali et al., 2021), while studies from Oman found many IBV genotypes, including 793/B, in backyard flocks (Al-Malki et al., 2021). These geographical results are consistent with Iranian data suggesting unvaccinated backyard hens have a molecular prevalence of 41.3% and contain a variety of lineages, such as 793/B, QX, and Variant 2 (Shokri et al., 2018). Further evidence of possible viral transmission between domestic and wild hosts comes from the discovery of gamma-coronaviruses in asymptomatic free-ranging chickens and wild ducks along Iran's Caspian coast (Rezaee et al., 2020Together, these findings show that backyard chickens are significant IBV reservoirs (Boroomand et al., 2018a; Shokri et al., 2018) and that their proximity to commercial farms—along with a lack of biosecurity—poses a persistent risk of viral introduction to industrial poultry operations (Ghalyanchilangeroudi et al., 2019; Haji-Abdolvahab et al., 2019), highlighting the necessity of integrated surveillance across the region's production systems (Gonzales-Viera et al., 2021; Legnardi et al., 2020).

This review has limitations that should be considered when interpreting the results. First, the extracted dataset did not include the denominator (sample size) and numerator (number positive) for each study; therefore, we could not compute prevalence estimates or undertake quantitative synthesis. Second, reporting of poultry population characteristics (e.g., age, production type, and vaccination history), sampling strategy, and genotyping methods was inconsistent in several studies, which may bias the apparent distribution of lineages. To enable stronger inference and future meta-analyses, Iranian primary studies should adopt standardized reporting that includes sample size, number positive, confidence intervals, precise geographic location, vaccination status, and sequence-based lineage assignment, consistent with modern systematic review reporting standards. Despite these limitations, the overall evidence indicates sustained circulation and diversity of IBV in Iran. Strengthening routine molecular surveillance and aligning vaccination strategies with circulating lineages may improve disease control and reduce production losses. Future work should also prioritize systematic sequencing of the S1 gene (and, where possible, whole-genome sequencing) to characterize recombination events better and inform regional vaccine updates.

## Conclusions and applications

1. The ongoing circulation of several IBV lineages, such as GI-1 (Massachusetts), GI-13 (793/B), GI-19 (QX-like), and GI-23 (Variant-2/IS-1494/06-like), in Iranian poultry is confirmed by this systematic review. The current vaccines (H120, H52, 4/91) exhibit inadequate cross-protection because of their low genetic homology (72–83%) with field isolates. Based on the currently available data, we advise combination vaccination (Mass + 793/B), with a focus on the native Razi 793/B.08IR vaccine, especially in areas where Variant-2/GI-23 predominates.
2. Available studies, mainly based on PCR-based detection and S1 gene characterization, have repeatedly identified major IBV lineages including Massachusetts, 793/B, QX-like, and Variant-2/IS-1494/06–like strains, suggesting the co-circulation of multiple variants within Iranian poultry populations and reflecting the complex evolutionary dynamics of IBV.
3. However, variations in sampling strategies, reporting formats, vaccination histories, and lineage assignment methods have limited accurate comparison among studies and restricted comprehensive assessment of IBV diversity and distribution.
4. Future investigations should implement standardized molecular surveillance approaches, including systematic S1 sequencing and, where feasible, whole-genome sequencing, to improve lineage tracking, clarify viral evolution, and support vaccination programs adapted to locally circulating strains. Future control efforts should prioritize establishing a national molecular surveillance network, systematic S1 sequencing, and periodic vaccine–field strain matching to guide evidence-based vaccine updates.
5. Integrated control strategies combining targeted vaccination, enhanced biosecurity, and continuous molecular monitoring will be essential to reduce the impact of IBV and improve the sustainability of Iranian poultry production. Future primary studies should adhere to standardized reporting guidelines, such as STROBE-Vet and PRISMA, and consistently report sample sizes, number of positive samples, confidence intervals, and complete molecular characterization data (including S1 gene sequences and phylogenetic analyses) to enable quantitative synthesis (meta-analysis) in future systematic reviews.
6. Future systematic reviews in this field should prospectively register their protocols in PROSPERO or the Open Science Framework and clearly report all search parameters, including database-specific search strategies, to enhance methodological transparency and reproducibility.

## Author Contributions

Author 1 (Arash Ghalyanchi Langeroudi) contributed to data analysis, the writing of the initial draft, and the review and editing of the manuscript.

Author 2 (Hamideh Najafi) contributed to the literature search and collection of articles, as well as the review and editing of the manuscript.

Author 3 (Kimia Malekahmadi) participated in the collection of articles and data extraction; additionally, she played a role in preparing the initial draft of the article.

Author 4 (Sayyad Khanizadeh) contributed to the formal data analysis and the preparation of the initial draft.

Author 5 (Iman Pouladi) contributed to data collection and extraction. He also oversaw the research project as a whole. Furthermore, he was responsible for preparing both the initial draft and the revised version of the manuscript.

## Disclosures

The authors declare that they have no conflicts of interest.
